# Profiling Particles of Sahara Dust Settled on the Ground by a Simplified Dynamic Light Scattering Procedure and Sedimentation

**DOI:** 10.3390/ijerph20064860

**Published:** 2023-03-09

**Authors:** Dan Chicea, Sorin Olaru

**Affiliations:** 1 Department of Environmental Sciences, Lucian Blaga University of Sibiu, Dr. Ion Ratiu Str., no 5-7, 550012 Sibiu, Romania; 2 Research Center for Complex Physical Systems, Faculty of Sciences, Lucian Blaga University of Sibiu, Dr. Ion Ratiu Str., no 5-7, 550012 Sibiu, Romania

**Keywords:** Saharan dust cloud, dynamic light scattering, sedimentation, particle sizing

## Abstract

Dust particles exist in the form of mineral aerosols and play a significant role in climate change patterns, while also having the potential to affect human health. The size of these particles is crucial, as it determines the atmosphere’s albedo. In the past few years, a Saharan dust cloud has moved and arrived above Romania during spring, followed by rain containing the dust particles, which are deposited on various objects. We collected these particles in an aqueous suspension and employed natural sedimentation to separate them by density. We then conducted a dynamic light scattering (DLS) experiment to analyze their size. Our DLS setup was straightforward, and the time series analysis involved evaluating the frequency spectrum of the scattered light intensity—also known as the power spectrum—filtering it, and fitting the expected Lorentzian line to it to determine the parameters and the average diameter of the suspended particles. We found that the dust particles had a continuous distribution, with the biggest particles having a diameter around 1100 nm. The results obtained from the combination of sedimentation and DLS are consistent with reports on the size of Saharan dust particles in other regions of Europe.

## 1. Introduction

At the beginning of April 2022, a Saharan dust cloud moved and arrived above Romania’s territory [1], followed by a rain containing the dust particles (aerosols), which were deposited on objects. Dust aerosols are one of the major factors affecting the global climate system. It is estimated that roughly 57 % of mineral aerosols are produced in North Africa [2,3].

Atmospheric aerosols and their optical parameters are a major factor in global climate change estimation models [3]. Dust particles present in the atmosphere can have either a cooling or a warming effect, depending on the altitude of the layer, single-scattering albedo, and the albedo of the underlying surface. Reference [4] also states that over surfaces with a surface albedo greater than 0.3, mineral aerosols typically warm the atmosphere. Over oceans, forests, and dark surfaces with surface albedo smaller than 0.15, mineral aerosols have a cooling effect on the atmosphere. Reference [4] also states that for albedo values between 0.15 and 0.30, the effect depends on both the size distribution and the chemical composition. Consequently, the net radiative effect presents important regional variations, which partly explains the difficulty in estimating the magnitude and the type of (warming or cooling) effect of the radiative perturbation caused by the dust particles [4].

Moreover, the desert dust transported by air currents strongly contributes to air pollution, reducing air quality by increasing particulate matter concentrations, which affects human health, by increasing mortality risk [5,6,7]. The mean annual temperature in the Sahara is higher than 30 °C [5], making this region one of the hottest regions on the surface of the Earth. It extends from the Atlantic Ocean to the Red Sea, between sub-Saharan Africa in the South to Mediterranean North Africa in the North, being the largest desert in the world. Sandstorms usually occur under very strong winds generated by the large difference in air density between warm and cold air masses [8]. This usually appears in summer but might also occur in spring, as in 2022 [8].

The Saharan dust cloud is most frequently transported towards the west into the Atlantic Ocean [9,10,11]. Dust is also transported across the Mediterranean Sea and, on a few occasions, has traveled as northwards as the shores of the Baltic Sea [12,13,14,15,16,17]. In the spring of 2022, the Saharan dust cloud arrived over the territory of Romania, and dust was deposited by rain on surface objects [1].

References report that the dust particle size and chemical composition have been analyzed using different methods. Reference [18] used scanning electron microscopy (SEM) for particle sizing and particle size distribution (PSD) assessment, coupled with energy-dispersive X-ray spectrometry (EDX), to identify the chemical composition. The authors identified eight major particle-type classes with clay minerals comprising most of the particles. They also identified carbonates and quartz. According to reference [18], the dust aerosols also contained particles that were rich in iron and feldspar. These particles have a significant impact on the radiative properties of the dust aerosols. The work in reference [19] mentioned using the Passive Cavity Aerosol Spectrometer Probe (PCASP), and found that the aerosol size distribution contained most of the particles within the 0.1–2.0 μm diameter range. They also mentioned that this radius range contains most of the optically active particles and used radiometric measurements to derive the scattering and absorption coefficients of the aerosols.

Other methods used in assessing the PSD are the scanning mobility particle sizer (SMPS) [20] or a combination of SMPC with the optical particle counter [21], which investigated the aerosols diameter range from 10 nm up to 18 µm.

A deeper insight into the size of the desert aerosols is offered by reference [22], which mentions the aerodynamic, geometric, and optical diameters and describes the difference between them. The geometric diameter, defined as the diameter of a sphere with the same volume as the irregularly shaped dust particle [23], can be measured with a coulter counter. Other techniques make use of optical imaging techniques, as reported in [24]. The different diameters of the particle size can differ significantly for the same particle, as mentioned in [25].

The work reported here used an optical technique, named dynamic light scattering (DLS), to assess the size of the Saharan dust. DLS involves an analysis of the far interference field and is a well-established technique frequently used to assess the diameter of the particles, whether organic or inorganic, suspended in a liquid solvent [26,27,28,29]. The work described in this paper presents the results of DLS measurements that were performed on Saharan dust particles suspended in aqueous suspension. Moreover, sedimentation, which is a process that occurs naturally if a particle has a density bigger than the density of the fluid they are suspended in, was used in profiling the size of the suspended particles. The basic physics of DLS and sedimentation used in profiling particles are described further on in the Section 2 in detail. The results and discussion are presented in the subsequent section, followed by Section 4.

## 2. Materials and Methods

### 2.1. Saharan Dust

On April 4 and 5 of the year 2022 a Saharan dust cloud arrived above Romania’s territory [1]. Rain followed, containing the dust particles (aerosols), which were thus deposited on surface objects. As the cloud contained low concentrations of Saharan dust, its main effect was of dirtying car windshields and bodies, and the windows of houses. Such deposits were sampled on April 6th using a sterile bandage previously humidified in deionized water. The wet bandages were soaked in deionized water and squeezed, relieving the water that contained the dust particles suspended in it. All operations were performed using latex sterile gloves. We must mention here that the air naturally contains a small amount of local dust produced by the air currents that exist in any region. As the period of study had seen a relatively large amount of rain for about two weeks before the dust cloud arrived over the Romanian territory, most of the local dust was transported to the ground by rain. After several days of rain, the objects on the ground, such as cars—especially those with dark paint where dust is more noticeable—did not show any signs of dust buildup once the rain had ceased. This suggests that there was a minimal amount of local dust present in the air, if any at all. The dust that was transported to the ground by rain after the Saharan dust arrived above the territory was therefore mostly Saharan dust, with a very small amount of local dust—albeit in an amount that was not estimated. Hereafter, we name this dust the Saharan dust.

The suspension containing the Saharan dust particles was preserved at +2 °C in a refrigerator until it was analyzed using the combination of DLS and sedimentation, as is described later in this paper. The fluid obtained as described, the aqueous suspension, was stirred for 5 min using a magnetic stirrer, and a small amount of 1.5 mL was transferred in the transparent tube of the custom DLS device described in Section 2.2.

We should note at this point that the Saharan dust was lifted up in the atmosphere by the hot air currents; first transported over the Atlantic Ocean [9,10,11]; then over the western part of Europe [12,13,14,15,16,17]; and finally, transported over the Romanian territory. With this in mind, we understand that there was ample time for the giant dust particles to be deposited.

### 2.2. Dynamic Light Scattering

DLS is a well-established technique frequently used to assess the hydrodynamic diameter of the particles suspended in a liquid solvent. It became practically possible only after light sources with both a very small spectral linewidth and very good coherence became available and could be used for research [26]. Such a light source is a laser. Some of the first publications reporting results using the DLS technique are [27,28,29,30]. DLS is a fast optical procedure and only requires a small amount of sample.

Experimentally, the particles suspended in a liquid solvent are the target of the laser beam. The coherent incident light is scattered by each particle in the beam area in all directions, therefore producing the far interference field. As each location of the interference field contains information regarding the motion of each particle that scattered light, it is possible to assess information on the diffusion properties of the particles, and from it the dimension of the particles [30,31,32,33]. This is accomplished by recording a time series from a location of the far field, most often at a scattering angle of 90°, and by analyzing it to assess the diffusion coefficient; then, from here, the particles size distribution or the average hydrodynamic diameter of the suspended particles. 

The cumulants method [34] is one of the first methods used to analyze the DLS time series and is based on the autocorrelation function of a monomodal distribution. This method does not accurately describe multimodal systems. To solve this problem, the non-negative least squares (NNLS) analysis was introduced [35]. Other methods, which can be considered variants of NNLS, are CONTIN [36,37] and the Maximum Entropy algorithms [38,39].

CONTIN is based on the inverse Laplace transform [36,37]. It introduces regularization to decrease the number of mathematical structural items. The inverse Laplace transform, when applied to numerical data, is computation intensive [40] and requires filtering [41]. The inverse Laplace transform is an ill-posed mathematical problem and may lead to ambiguous results on numerical data, as with a DLS time series. CONTIN partially overcomes this by introducing regularization. The parameter used in this regularization has a significant influence on the solution. The best value selected for this parameter might not be the solution that is closest to the reality and can lead to completely incorrect solutions.

The maximum entropy method [38,39] is an improvement and consists of assigning entropy to the solutions. The solution with the maximum entropy is selected as the solution. This process is also computationally intensive.

There are also newer alternatives for processing DLS time series, based on the use of artificial neural networks (ANNs) [42,43]. Reference [44] reports on using an averaged scattered light intensity frequency spectrum as input for an ANN that estimates the average diameter of the particles in suspension, for particles with a diameter smaller than 350 nm. Reference [45] reports on the continuation of the work in [44] and extends the range of particle size up to 1200 nm; it also contains the autocorrelation of the DLS time series as input. Reference [46] extended the range of the suspended particle size up to 6000 nm. The ANN based procedures reported in [44,45,46] were hundreds [44] or thousands of times [45,46] faster than the procedures based on fitting an analytical function, whether on the frequency spectrum or on the autocorrelation of the DLS time series.

Despite being very fast, these alternatives based on ANNs do not offer any indication regarding the precision of the result, with respect to the accuracy of the monomodal approximation they are based on. An attempt to use DLS on particles in air as solvent was made, and the results are reported in [47]. However, the precision is not considerable, the main cause being the small angle and the relatively large error in measuring the angle caused by the diameter of the transparent tube containing the sample. Moreover, the concentration of particles required to perform DLS measurements on particles in air is quite large, as the concentration of particles in fumes is; therefore, this alternative is not suited for a very low concentration of particles in air produced by the Saharan dust cloud that arrived over the eastern part of Europe.

**Figure 1:**
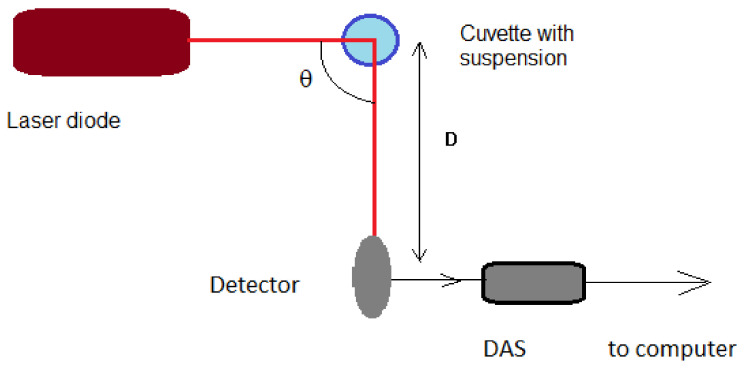
The schematic of the experimental setup, with view from above. The dark blue circle represents the cuvette and the light blue disc inside represents the aqueous suspension.

To overcome the uncertainty that might be generated by using an ANN-based alternative, and to overcome the possible problem of not having a sufficiently large particle concentration, a DLS time series data processing procedure that uses a fit of an analytical function to the experimental data was used in the work reported in this paper. Moreover, the procedure intended to simplify the experimental setup as much as possible and make it flexible, as reported in [44,45,46,47]; this is briefly presented further on. The experimental setup is presented in Figure 1.

The setup consists of a laser diode, the cylindrical cuvette containing the suspension, a data acquisition system (DAS), a detector and a computer to record the time series. The wavelength for a laser diode light beam is 635 nm and is one of the most frequently used. The laser diode worked in a continuous regime at a power of 15 mW. The DAS is common, with one channel used [48], and the data acquisition rate was 16,000 samples per second. The scattering angle θ is variable. A 90° angle was chosen as the scattering angle to record the DLS time series for the work reported here. The distance between the sample and detector can be adjusted so that the average speckle size will match the size of the detector (as close as possible), and it was chosen to be 10 cm. The transparent tube containing the aqueous suspension had a diameter of 1 cm.

The scattered light intensity time series (TS) recorded as described above were processed in several steps. First, the Fourier transform using the Fast Fourier Transform (FFT) algorithm [49,50] was used to compute the frequency spectrum of the light intensity, also named power spectrum (PS). The FFT algorithm works on sets of data containing a number of data points of the form 2^n^, where n is a natural number, which was chosen to be 19 for the TS recorded during measurements; therefore, the TS were 327,680 s long. It was possible to achieve such a relatively large length of the TS because the suspension was stable. It produced many frequency–amplitude pairs (262,145 pairs actually) [50], assuring a reliable fit of the Lorentzian function.

The second stage follows the original procedure [29,30] (previously employed in data processing [44,51,52,53]) of fitting the expected function for PS, which is the Lorentzian function (1), to the PS computed on the experimentally recorded TS, using a nonlinear fitting procedure. The fit produces the parameters a_0_ and a_1_.

(1) $$S\left(f\right)=a_{0}\frac{a_{1}}{{\left(2\pi f\right)}^{2}+a_{1}^{2}}$$

In (1), the a_0_ parameter performs the vertical scaling of the spectrum, but a_1_ depends on the diameter d of the particles in suspension, which act as scattering centers (SCs), as described by (2) [45,46,47]:

(2) $$d=\frac{2k_{B}{\mathrm{Tq}}^{2}}{3{\pi \eta a}_{1}}$$

In (2), q is the magnitude of the scattering vector, as described by Equation (3):

(3) $$q=\frac{4\pi n}{\lambda }\sin\frac{\theta }{2}$$

In Equations (2) and (3), k_B_ is Boltzman’s constant, n is the refractive index of the solvent, λ is the wavelength of the laser beam, T is the absolute temperature of the solvent, η is the dynamic viscosity of the solvent and θ is the angle used to record the TS, which is usually called the scattering angle [46].

The recording started immediately after the fluid was transferred into the cuvette, in an automated manner, with TS lasting for 34 s and a delay of 29 min and 26 s between them. The procedure lasted for 45.5 h. The TS were loaded in an array, keeping 2^19^ data in each one, and processed in batch mode, as described in Section 2.2, with the average diameters as output. 

**Figure 2:**
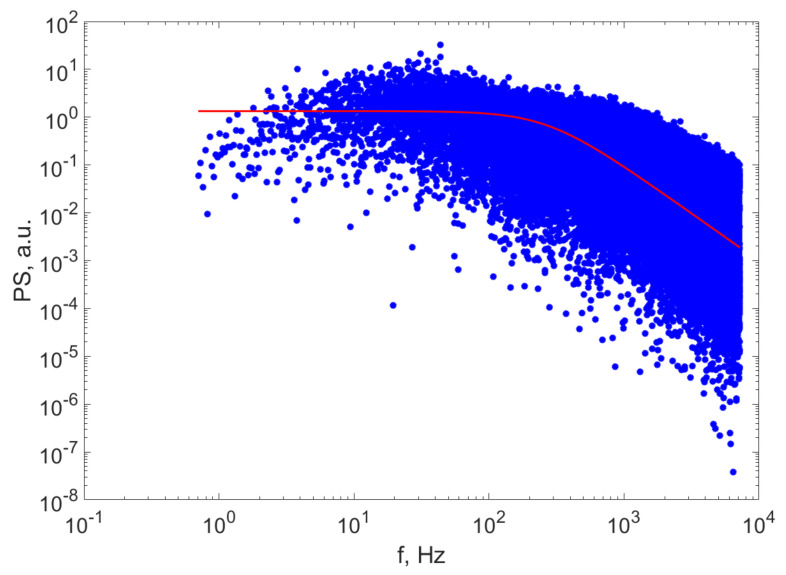
The PS of the scattered light intensity for a suspension (blue circles) and the Lorentzian fitted line for the suspension after (continuous line|) at 10.5 h of sedimentation.

The average diameter of the particles in suspension is calculated late in this paper using Equation (2). Figure 2 illustrates the PS computed values of the TS on the experimental TS and the fitted Lorentzian line.

Examining the plot of the computed PS data and the fit of the Lorentzian line, we notice that the line sufficiently described the computed data, which confirms the hypothesis that the distribution can be approximated as being monomodal. 

### 2.3. Error Calculation for the DLS Diameters

The relative error when assessing the particle radius R can be estimated by replacing Equation (3) in Equation (2), and by writing the logarithm of d, as in Equation (4):

(4) $$d=\frac{32{\pi k}_{B}n^{2}}{3{\eta a}_{1}\lambda^{2}}{\mathrm{Tsin}}^{2}\frac{\theta }{2}$$

If we consider all the constants to be grouped as one factor, the differential of that factor will be null. If we consider that the quantities that we measured—which, therefore, were sources of errors—were the thermodynamic temperature T and the measuring angle θ, the logarithm of d is:

(5) $$\ln\left(d\right)=\ln\frac{32{\pi k}_{B}n^{2}}{3{\eta a}_{1}\lambda^{2}}+\mathrm{lnT}+2\ln\left(\sin\frac{\theta }{2}\right)$$

If we differentiate Equation (5) and consider dT and dθ to be the experimental errors in measuring those quantities, under the assumption of the most unfavorable situation when errors sum, we obtain Equation (6):

(6) $$\varepsilon_{d}=\frac{\Delta d}{R}=0+\frac{\Delta T}{T}+\frac{1}{\tan\left(\frac{\theta }{2}\right)}\Delta \theta$$

The error in measuring the temperature was 1 K for temperature T, which was 20 °C, hence 293.15 K. The detector–tube distance was 10 cm, and the diameter of the tube was 1 cm, which makes Δθ:

(7) $$\Delta \theta =2\mathrm{atan}\frac{0.5d_{\mathrm{tube}}}{D}$$

The relative error calculated with Equation (6) was therefore 10.3%. Consequently, the error bars of the diameters were calculated using this value of the relative error.

The error is relatively large but is still in line with the purpose of the work described in this paper, to use a simple setup and data processing procedure for assessing the diameter of the particles suspended in aqueous suspension.

### 2.4. Particle Size Separation by Sedimentation

**Figure 3:**
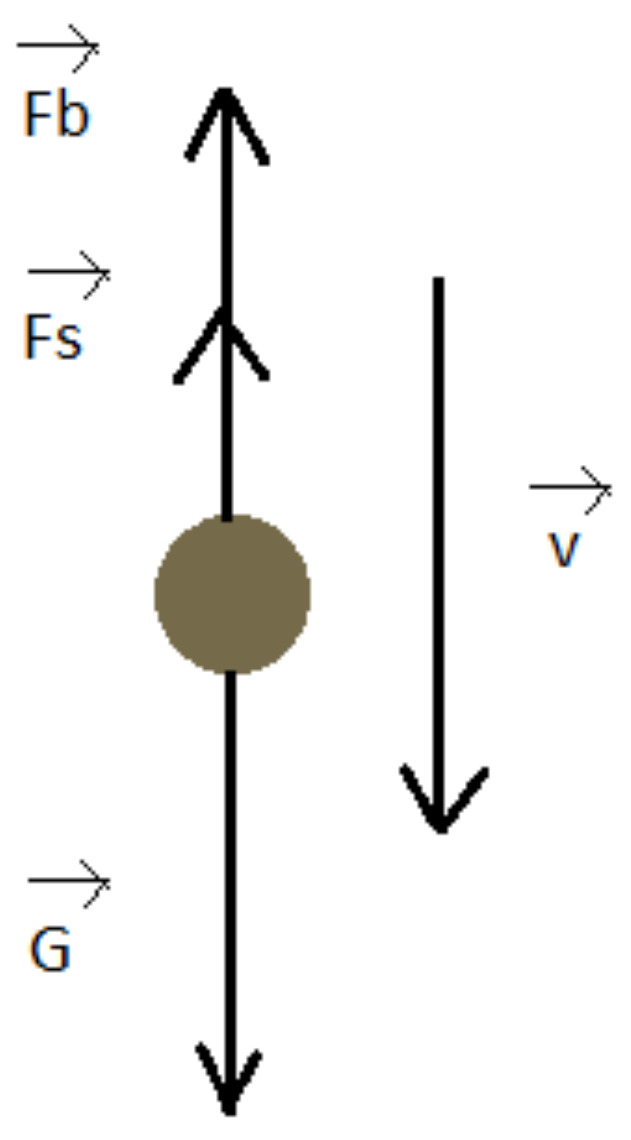
The forces exerted on a particle in a fluid.

If a particle is suspended in a fluid, it is subject to the action of three forces: gravity, buoyant force and the Stokes drag, because the motion of very small particles takes place in the laminar regime [54]. If d is the diameter of the suspended particle, the buoyant force Fb, gravity G and Stokes drag Fs are illustrated in Figure 3 and are expressed as:

(8) $$F_{b}=\frac{\pi }{6}d^{3}\rho_{0}g$$

(9) $$G=\frac{\pi }{6}d^{3}\rho g$$

(10) $$F_{s}=3\pi \eta \mathrm{dv}$$

In Equations (8)–(10), d is the diameter of the particle, η is the dynamic viscosity of the fluid, ρ and ρ_0_ are the densities of the particle and of the fluid, g is the gravitational acceleration and v is the velocity of the particle in fluid. If the fluid density is smaller than the density of the particle, the velocity is pointing downwards, and the particles undergo sedimentation. The fluid was deionized water with a density of 1000 kg/m^3^, and the density of the particles was considered to be 2648 kg/m^3^, which is the density of SiO_2_.

In Equations (8)–(10), the gravity and the buoyant force are constant, but the Stokes drag increases with the velocity up to the point that the vector sum of forces is null; therefore, the velocity remains constant and equal to a limit velocity v_l_, as described by Equation (11):

(11) $$v_{l}=\frac{\left(\rho -\rho_{0}\right)d^{2}g}{18\eta }$$

**Figure 4:**
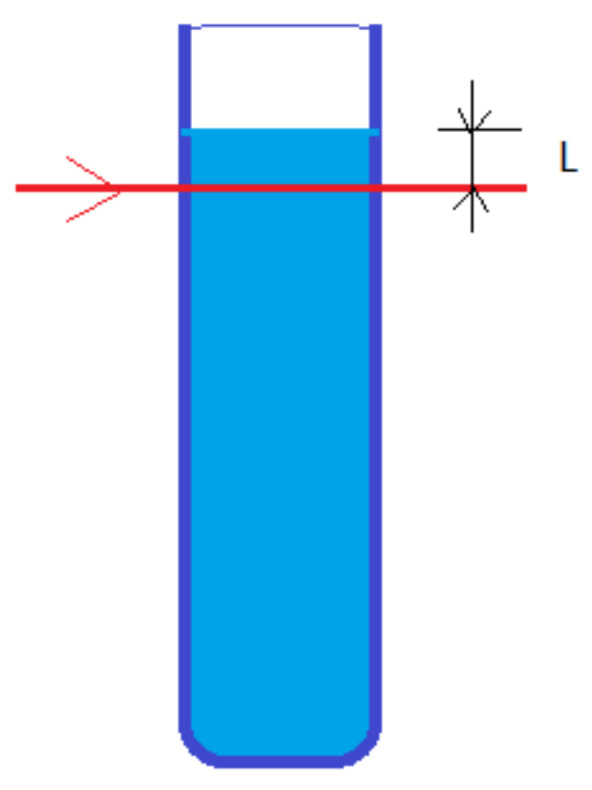
The sedimentation tube and the laser beam.

We notice that the limit velocity strongly depends on the particle size, as it is proportional with the square of the diameter. Bigger particles sediment faster than smaller particles, and this can be used to separate particles by their size in a simple and effective manner. If we consider a vertical tube and a laser beam at a distance L from the free liquid surface, as in Figure 4, after time *t* from pouring the fluid in the beam area, only particles that have a limit velocity smaller than *v_m_* in Equation (12) will remain; therefore, they will have a diameter smaller than *d_max_* described by Equation (13).

(12) $$v_{m}\left(t\right)=\frac{L}{t}$$

(13) $$d_{\max}=3\sqrt{\frac{3{\eta v}_{m}}{\left(\rho -\rho_{0}\right)g}\;}=\sqrt{\frac{3\eta L}{\left(\rho -\rho_{0}\right)\mathrm{gt}}}$$

The free surface level of the fluid was carefully adjusted inside the sedimentation tube to position the laser beam directly under the free surface, whilst avoiding its reflection on the curved surface. As water is wetting glass, the free surface is not a plane surface but has a concave shape. With the caution described above, the L distance was estimated to be 0.15 mm.

## 3. Results and Discussion

**Figure 5:**
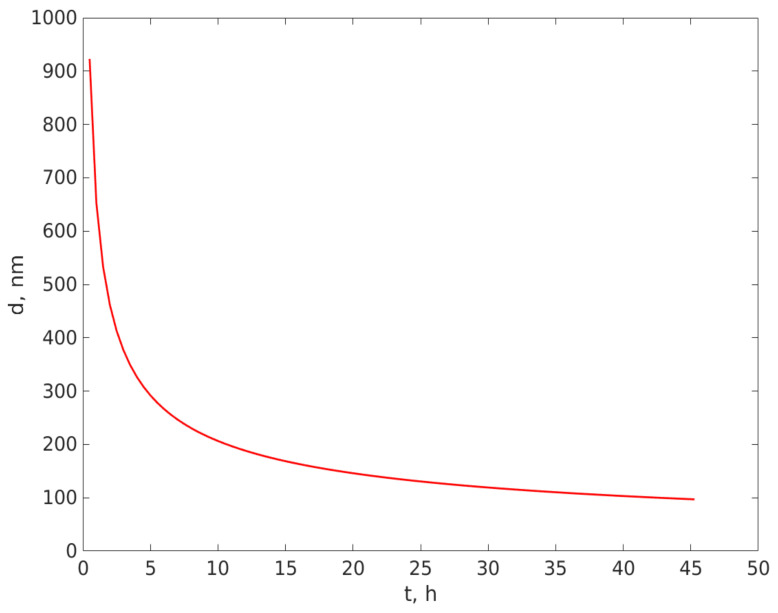
The maximum diameter, in nm, of the particles still in suspension in the laser beam area versus time t, in hours.

A plot of the largest diameter of the particles remaining in the beam area versus the time elapsed from pouring the suspension, calculated using Equation (13) and distance L, as mentioned above, is illustrated in Figure 5.

Examining Figure 5, we notice that for such a small value for L, the variation of the diameter of the particle remaining in suspension is quite fast over a time interval of tens of hours, and this enables an estimation of the type and the size of particles suspended in fluid using this very simple sedimentation experiment and the custom DLS particle sizing procedure. If the particle density is larger than the fluid density, the curve presents a decrease in the particle size due to sedimentation, as illustrated in Figure 5. On the contrary, if the particle density is smaller than the fluid density, the curve would present an increase in the particle size over time, because the particles would be moving upwards in the tube, as the buoyant force would be greater than the gravitational force.

**Figure 6:**
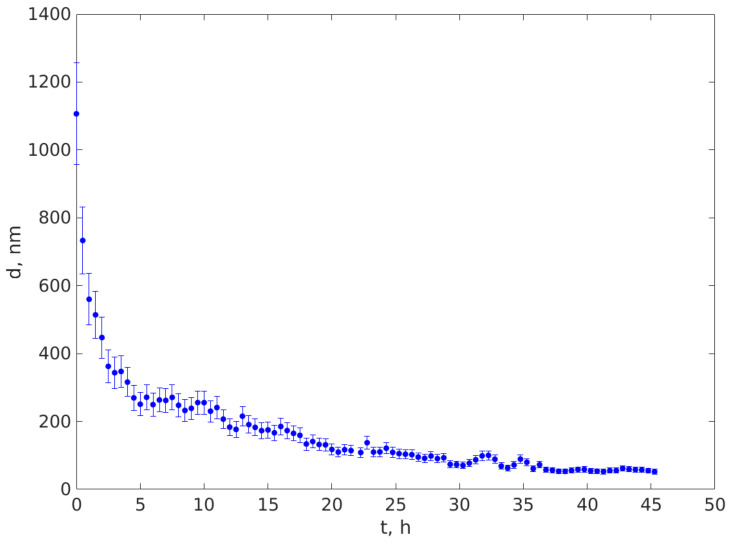
The variation of the average diameter of the particles that remained in the beam area during sedimentation versus the elapsed time.

Figure 6 illustrates the variation of the average diameter of the particles remaining in suspension in the beam area with the time elapsed from depositing the suspension in the tube and the beginning of the sedimentation. 

Upon examining Figure 6, we notice that the average diameter of the particles in the upper part of the sedimentation tube where the beam area was located was around 1100 nm at the beginning of the sedimentation procedure and decreased quickly during the first 4.5 h to around 300 nm, followed by a slower decrease during the subsequent hours of sedimentation. The apparent increases in the diameter at timed t = 10 h and t = 32.5 h are, most likely, a fitting artefact, produced by using the same start parameters for all the time series. After 45 h of sedimentation in the natural gravitational field, the largest diameter of the particles in suspension in the beam area was 50 nm. We should note at this point that the diameters would have continued to decrease but recording was stopped after 45 h; this is because it appears unpractical for an experiment to last for weeks, simply to make evident the presence of particles smaller than 30 nm, but most likely, the distribution also contains particles smaller than 50 nm.

We also notice that the excellent resemblances of the trend in Figure 6 with the estimated diameter remaining in the beam are at L = 0.15 mm, calculated using Equation (13) and illustrated in Figure 5. If the particle density is greater than the fluid density (Section 2.4), the curve presents a decrease in the particle size due to sedimentation, as illustrated in Figure 5. The very good resemblance of the trend and the values in Figure 5 and Figure 6 are a strong indication that the suspended particles that were harvested from rain deposition had a density that was greater than the water density. The very good resemblance of the values in the two curves indicate that the density of the particles is very close to the density used in calculating the diameters plotted in Figure 5, which was 2648 kg/m^3^; therefore, the deposited particles are most likely SiO_2_. 

We should also mention that the diameter computed using the DLS procedure is derived from the diffusion coefficient; therefore, it is the hydrodynamic diameter, not the physical diameter. It can be realistically understood as the diameter of particles that diffuse as spheres with the same diameter, regardless of their shape, which can be rod, ellipsoid or an irregular shape.

Moreover, the fit of the expected Lorentzian line on the PS calculated on the experimentally recorded TS is very good, as can be seen in Figure 2, which confirms that the approximation that the size distribution of the particles is monomodal is realistic. We should bear in mind that this approximation is consistent with the fact that the intensity of the scattered light is proportional to the sixth power of the diameter of the scattering particles at a certain angle [54], if the diameter is smaller than the wavelength, which suggests that the diameter computed as described above is not the average diameter in the conventional way, but is rather the average of the diameters of the biggest particles in suspension in the beam area. These aspects are presented in detail in [55], where a comparison is presented on the diameters of the particles from the same batch assessed using DLS, X-ray diffraction and atomic force microscopy measurements.

## 4. Conclusions

A combination of particle separation by sedimentation in the normal gravitational field, and an analysis using a custom DLS device and data processing for monomodal particles size distribution, was used to analyze the Saharan aerosol particles. This method involved harvesting the particles deposited by rain on a clean surface and analyzing the suspension of such particles in deionized water, which, to our knowledge, has not been reported so far.

Larger particles sediment faster, causing the larger diameter of the particles remaining in suspension at a certain level to decrease over time if the density of the particles is bigger than the fluid density (water in our experiments), and to increase over time if the density of the particles is smaller than the density of the fluid. We found that the size of the particles, measured using the DLS procedure described in Section 2, at a level lower than the free surface of the fluid, decreased over time. Further, the plot of the DLS diameters over time, illustrated in Figure 6, sufficiently resembles the curve of the calculated diameters of SiO_2_ particles presented in Figure 5, which is a strong indication that the particles transported over Eastern Europe from the Saharan sand storm in the spring of 2022 were SiO_2_ particles. Moreover, the plot in Figure 6 indicates that the particles had a continuous distribution, with the biggest particles having a diameter around 1100 nm and down to 50 nm, as measured, but containing particles smaller than 50 nm, as explained in Section 3.

The diameter assessed using the DLS procedure presented in Section 2 should be viewed as the hydrodynamic diameter, which is the diameter of spherical particles that diffuse as the particles in suspension; therefore, the procedure does not indicate the actual shape of the particles. Moreover, the scattered light intensity for small particles, as found in suspension, increases quickly with the diameter, and the interference field is dominated by the light scattered by the biggest particles, as explained in [54]. The diameter assessed by the DLS procedure should be viewed as the average of the biggest particles found in suspension at the beam level; therefore, the combination of sedimentation and the simple DLS procedure can be used to carry out profiling of the particles in suspension. 

The results reported in this work are consistent with the results regarding the Saharan dust particle size distribution presented in [56], with most of the particles smaller than 1000 nm. The very good resemblance is probably related to the large distance traveled by the particles transported by air currents before being sampled, to the Eastern Atlantic [56] and over the Eastern part of Europe, after first being transported over the western part of Europe, as reported here.

Reference [18] reports on particle size distribution assessed over the southern part of Morocco, over the Saharan desert, where the hot air currents lift dust particles up into the atmosphere. The authors report in [18] that most of the particles, with respect to volume size distribution, can be found in the 2–20 µm diameter range. This is not surprising, because the strong ascending hot air currents formed by the high temperature of the sand lifted bigger particles into the atmosphere. Bigger particles sediment faster than smaller particles, regardless of the fluid they are suspended in, as revealed by Equation (11);, therefore coarse and giant particles sedimented after exiting the ascendent hot air currents, while smaller particles sedimented much slower, which explains why the longer the particles travel, the more the size distribution is shifted towards smaller diameters. The results presented in this work are also consistent with the report in [21], where the particles were found to be in the 10–1000 nm diameter range, as measured in France. Again, this can be an indication that giant and coarse particles sedimented before the air current transported them above the observation location. Similar results, this time with respect to concentration, are reported on the particle size distribution of the Saharan dust cloud measured above Portugal [57], where most of the particles were found in the same 10–1000 nm diameter range, as in the work presented here. The particle diameter ranges measured in this work are consistent with the results presented in reference [19], and these references are just a few of the reports on this subject.

The work reported here presents a simple method, a combination of sedimentation and DLS, that can be used to assess the particle size of dust that travels by rain to the ground’s surface and is collected in sterile conditions, as sustained by the consistency of the results with those obtained using the consecrated methods reported in the literature.

## Data Availability

Not applicable.
